# Identification of a mimotope for circulating anti-cytokeratin 8/18 antibody and its usage for the diagnosis of breast cancer

**DOI:** 10.3892/ijo.2012.1679

**Published:** 2012-10-26

**Authors:** CHANG-KYU HEO, HAI-MIN HWANG, AH RUEM, DAE-YEUL YU, JU YEON LEE, JONG SHIN YOO, IN GYU KIM, HYANG SOOK YOO, SEJEONG OH, JEONG HEON KO, EUN-WIE CHO

**Affiliations:** 1 Cancer Biomarkers Development Research Center; 2 University of Science and Technology; 3 Aging Research Center, Korea Research Institute of Bioscience and Biotechnology;; 4 Division of Mass Spectrometry Research, Korea Basic Science Institute;; 5 Department of Radiation Biology, Environmental Radiation Research Group, Korea Atomic Energy Research Institute, Daejeon;; 6 Department of Surgery, College of Medicine, The Catholic University of Korea, Inchon, Republic of Korea

**Keywords:** autoantibody biomarker, anti-cytokeratin 8, 18 antibody, breast cancer diagnosis, mimotope enzyme-linked immunosorbent assay

## Abstract

A novel circulating tumor-associated autoantibody, K94, obtained from a hepatocellular carcinoma (HCC) mouse model was characterized. The target antigen of K94 autoanti-body was expressed in various tumor cell lines including liver cancer, and its secretion was detectable using MCF-7 breast carcinoma cells. Proteomic analysis revealed that the protein bands reactive to K94 included cytokeratin (CK) 8 and 18, which are known to be related to tumorigenesis and form a heterotypic complex with each other. However, K94 showed no activity toward CK8 or CK18 separately. The epitope of the K94 antibody was only presented by a complex between CK8 and CK18, which was confirmed by analysis using recombinant CK8 and CK18 proteins. To formulate an assay for anti-CK8/18 complex autoantibody, a mimotope peptide reactive to K94 was selected from loop-constrained heptapeptide (-CX_7_C-) display phage library, of which sequence was CISPDAHSC (K94p1). A mimotope enzyme-linked immunosorbent assay (ELISA) using phage-displayed K94p1 peptide as a coating antigen was able to discriminate breast cancer (n=30) patients from normal subjects (n=30) with a sensitivity of 50% and a specificity of 82.61%. CA15.3 was detected at very low levels in the same breast cancer subjects and did not discriminate breast cancer patients from normal subjects, although it is a conventional biomarker of breast cancer. These results suggest that a mimotope ELISA composed of K94p1 peptide may be useful for the diagnosis of breast cancer.

## Introduction

Components of body fluids are ideal biomarkers for disease diagnosis, because of their simplicity of detection. Serum has long been considered as a rich source for biomarkers and a large number of serum cancer biomarkers (i.e., AFP, PSA, CEA and CA15.3) have been proposed. However, serum protein biomarkers are often not sensitive enough to be used for screening and early diagnosis because their levels reflect tumor burden (1). Circulating autoantibodies against tumor-associated antigens are associated with cancer (2,3). Unlike the traditional tumor markers, serum autoantibodies to tumor antigens are detectable even when the tumor is very small, which makes them potential biomarkers for early cancer diagnosis (4). Moreover, the advantages of simple detection of autoantibodies in sera using target antigen and secondary reagents, in contrast to serum protein markers whose detection needs two different monoclonal antibodies, make it easy to construct a multiplex tumor-associated autoantibody assay.

Two main techniques have facilitated identification of many tumor-associated autoantibodies, serological identification of antigens by recombinant DNA expression (SEREX) (5,6) and serological proteome analysis (SERPA) (7,8). Recently, reverse phase protein lysate microarray has also facilitated the discovery of novel autoantibody biomarkers (7,8). These techniques are expected to be powerful tools for displaying thousands of candidate antigens at once and allowing analysis of many kinds of autoantibodies in patients’ sera simultaneously. However, patients’ sera are mixtures of hundreds of autoantibodies, and the amounts of each autoantibody are not comparable to each other. This makes the discovery of tumor-associated auto-antibodies biased toward the most abundant ones. For these reasons, tumor-associated antibodies discovered using these techniques are not so diverse and useful, despite the expectation that hundreds of tumor-associated autoantibodies are present in patients’ sera (9).

To identify tumor-associated autoantibodies separately, we constructed a B-cell hybridoma pool using splenocytes derived from an H-ras12V hepatocellular carcinoma (HCC) mouse model (10,11) and stable B-cell hybridoma clones which produce tumor-associated autoantibodies were selected according to their reactivity to human tumor cells. In our previous study, one of these tumor-associated autoantibodies was analyzed and identified as anti-fatty acid synthase (FASN) antibody. In addition, a diagnostic method for measuring anti-FASN autoantibody has been formulated using its mimotope which were screened from a cyclic peptide display phage library, and performed successfully for HCC diagnosis with a sensitivity of 96.55% and a specificity of 100% (10).

In this study, K94 monoclonal antibody, a tumor-associated autoantibody purified from another B-cell hybridoma clone derived from a mouse model of HCC, was analyzed and identified as an anti-cytokeratin (CK) 8/18 complex antibody. Also its mimotope was screened from a cyclic peptide display phage library, which corresponded to a conformational structure comprised of CK8 and CK18. Mimotope ELISA measuring CK8/18 complex antibody was performed using sera of tumor patients and showed to be useful for the diagnosis of breast cancer, but not for liver cancer.

## Materials and methods

### Cell lines and serum samples

The cell lines were obtained from the American Type Culture Collection (ATCC) and cultured in DMEM or RPMI-1640 supplemented with 10% fetal bovine serum. All cell lines originated from humans, except Hepa-1c1c7, which is a mouse hepatoma cell line, and HT22, which is a mouse hippocampal cell line. Human HCC or breast cancer serum samples were collected from Catholic Hospital in Inchon. The use of blood samples was approved by the ethics committee of Catholic Hospital. Normal serum samples were collected from volunteers or patients without cancer. Serum samples were kept at −70°C until use. Serum-free conditioned media were prepared from 70–80% confluent tumor cells, which were cultured for 48 h without fetal bovine serum.

### Preparation of K94 monoclonal autoantibody

B-cell clones secreting monoclonal antibody reactive to hepatoma cells were selected as described previously (10). K94, an autoantibody secreted from one of these clones, was analyzed in this study. The isotype of each autoantibody was determined using an isotyping kit (Pierce Protein Research Products, Rockford, IL). For preparation of K94 autoantibody, ascites fluid was produced and used for antibody purification using protein L-agarose (Pierce Protein Research Products).

### Flow cytometric analysis

Cells were fixed and permeabilized with BD Cytoperm/Cytofix solution (BD Biosciences, San Jose, CA), followed by incubation with primary antibody solution (hybridoma cell culture media or purified antibody) at 4°C for 40 min. Cells were washed and stained with anti-mouse Immunoglobulin (Ig) goat (Fab′)_2_-FITC (Abcam, Cambridge, MA). The stained cells were analyzed by FACScalibur (BD Biosciences) and data were analyzed using CellQuest software (BD Biosciences).

### Western blot analysis

Cell lysates were prepared as previously described (10) and protein concentration was determined by the Bradford method (Bio-Rad, Benicia, CA). Serum-free conditioned media from each cell line were concentrated and used for analysis. Equal amounts of protein (50 *μ*g) were resolved by SDS-PAGE and transferred onto a polyvinylidenedifluoride (PVDF) membrane (Millipore, Billerica, MA). The membranes were probed with K94 monoclonal antibody (mAb) or anti-CK8 or CK18 antibodies (Abcam). Positive bands were detected by horseradish peroxidase (HRP)-linked anti-mouse IgGAM antibody (Abcam) and other corresponding secondary reagents, followed by enhanced chemiluminescence reagents (GE Health Care Life Sciences, Pittsburgh, PA).

### Identification of K94 autoantigen

For partial purification of target proteins against K94 autoantibody, 500 ml of serum-free conditioned media from MCF-7 cells were concentrated using Amicon ultracentrifugal filters (Millipore) and fractionated using Hitrap-Q HP ion exchange columns (GE Health Care Life Sciences). Fractionation was performed with a linear gradient from 0 to 1 M NaCl dissolved in 10 mM phosphate buffer (pH 7.4) and positive fractions containing K94 antigen were confirmed by western blot analysis. Selected fractions containing K94 antigen were then pooled, concentrated and separated on 10% SDS-PAGE, followed by western blot analysis or Coomassie Blue staining. The Coomassie-stained bands corresponding to proteins reactive to K94 antibody were excised and used for in-gel digestion. Protein identification using in-gel protein digest was performed by using Nano-LC/ESI-MS/MS as described by Lee *et al*(12).

### siRNA transfection

To confirm whether the identified proteins (CK8 and CK18) were K94 antigens, MCF-7 or HT-29 cells were transfected with siRNA targeting CK8 or CK18 (Bioneer Corporation, Daejeon, Korea) using Lipofectamine RNAiMAX reagent (Invitrogen, Carlsbad, CA) and RT-PCR, flow cytometric and western blot analysis were performed 72 h after transfection. The sequences of siRNA targeting CK8 or CK18 were as follows; CK8 sense: 5′-CCG CAG UUA CGG UCA ACC A(dTdT)-3′, CK8 antisense: 5′-UGG UUG ACC GUA ACU GCG G(dTdT)-3′, CK18 sense: 5′-CUC ACA GAG CUG AGA CGU A(dTdT)-3′ and CK18 antisense: 5′-UAC GUC UCA GCU CUG UGA G(dTdT)-3′.

### RT-PCR

Total RNA was extracted from cells using Qiagen RNA extraction kit (Qiagen, Valencia, CA) and the first-strand cDNA was synthesized using Superscript III (Invitrogen). RT-PCR was performed using the following primer pairs; forward primer: 5′-ATG GAC AAC ATG TTC GAG AG-3′, reverse primer: 5′-CAG AGA TCT CAG TCT TTG TG-3′, for CK8, and forward primer: 5′-AGA GAC TGG AGC CAT TAC TTC-3′, reverse primer: CAA CCT CAG CAG ACT GTG TG-3′, for CK18.

### Recombinant CK8, CK18 and truncated CK18 proteins

For the preparation of CK8, CK18 and truncated CK18 proteins, corresponding sequences were amplified by PCR with unique primers (Table I) using cDNA prepared from MCF7 cells and nPfu DNA polymerase (Enzynomix, Seoul, Korea). PCR products were ligated into a pET29a(+) vector using suitable restriction sites, such as NdeI/SalI for CK8 and EcoRI/XhoI for CK18 and truncated CK18 proteins. The ligation mixtures were transformed into *E. coli* strain DH5α. Positive transformants were selected by restriction digestion and sequencing. Correct construct plasmids were transformed into BL21(DE3) cells and manipulated following conventional methods for preparation of recombinant proteins. The protein expression was induced by adding 1 mM isopropyl β-D-1 thiogalactopyranoside for 4 h. CK proteins, expressed as inclusion bodies in *E. coli*, were pelleted and solublized in 8 M urea in phosphate-buffered saline (PBS). After brief sonication, the supernatants from the inclusion bodies were used as recombinant CK proteins.

### Cytokeratin heterotypic complex formation

Western blot analysis of heterotypic CK8/18 complexes were performed following the method of Ditzel *et al*(13) with some modifications. Intact CK8, CK18 and truncated CK18s were separated by SDS-PAGE and transferred to PVDF membranes. After blocking with 5% skim milk in Tris-buffered saline supplemented with 0.1% Tween-20 (TBST) for 1 h at room temperature (RT), the membranes were incubated with 40 *μ*g of full length CK8 or CK18 proteins per ml of 4 M urea for 1 h at RT. The membranes were washed three times with TBST, incubated with 2 *μ*g of K94 autoantibody in 10 ml of TBS with 5% skim milk for 2 h at RT, and developed as described above. For enzyme-linked immunosorbent assay (ELISA), recombinant proteins (CK8, CK18 or truncated CK18 proteins) in 8 M urea were serially diluted with PBS to 0.5 M urea through four steps to allow renaturation of protein secondary structure and complex formation between CK8 and CK18 or truncated CK18. After complex formation between CK8 and CK18 or truncated CK18, mixtures were coated onto 96-well ELISA plates at a concentration of about 1 *μ*g/well (Maxisorp; NUNC, Thermo Scientific, Rochester, NY) and incubated at 4°C for 16 h. The plates were blocked with 5% skim milk in TBST at RT for 1 h. Diluted K94 auto antibodies, from 0.06 to 0.48 *μ*g per well in blocking buffer, were then incubated in these wells for 90 min. After washing with TBST, the wells were incubated with HRP-linked anti-mouse IgGAM antibody (1:2,500, diluted in blocking buffer, Abcam). Visualization was performed with 3, 3′, 5, 5′-tetramethylbenzidine (TMB, Pierce) at 100 *μ*l per well. After sufficient color development, 100 *μ*l of 2 M sulfuric acid was added to stop the reaction. The absorbance of each well was read with a plate reader (Molecular Devices, Downingtown, PA) at 450 nm.

### Immunofluorescence microscopy

Cells were plated onto 18×18 mm glass coverslips in 6-well plates and treated with BD Cytofix/Cytoperm solution at 4°C for 40 min. Fixed and permeabilized cells were incubated with primary antibodies (K94 or anti-CK8 and anti-CK18) which were diluted to 1 *μ*g/ml with BD Cytoperm/wash solution. After 1 h of incubation at 4°C, cells were washed and incubated with FITC-conjugated anti-mouse immunoglobulin F(ab′)_2_ antibody at 4°C for 1 h. Coverslips were mounted with Vectashield mounting medium containing 4′ 6-diamino-2-phenylindole (DAPI) (Vector Laboratories, Burlingame, CA) and analyzed using a Zeiss LSM510 Meta microscope (Carl Zeiss Micro Imaging, Thornwood, NY).

### Panning the phage library against K94 antibody

For selection of the mimotope specific to K94 autoantibody, the phage display random cyclic peptide library, Ph.D.-C7C™ (New England Biolabs, Ipswich, MA), was used following the manufacturer’s instructions (10). Panning was repeated five times, and sequencing of selected mimotope phages was performed following the manufacturer’s instructions.

### Phage ELISA

Phage ELISA was performed as described previously (10). ELISA results were evaluated by a receiver operating characteristic (ROC) curve, using Prism 5 software (GraphPad Software, La Jolla, CA).

## Results

### Target antigen of K94 tumor-associated autoantibody in various human tumor cell lines

**Figure 1.**
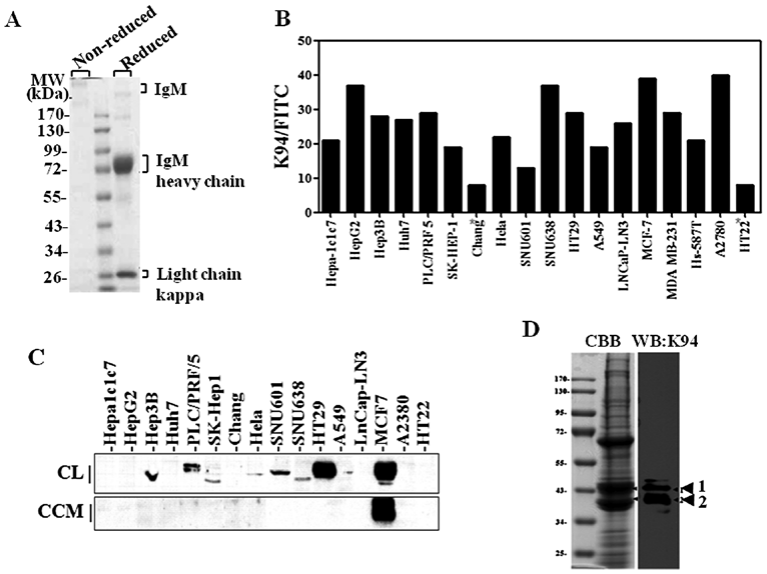
Target antigen of K94 tumor-associated autoantibody in various human tumor cell lines. (A) Purified K94 monoclonal antibody separated on non-reducing or reducing 10% SDS-PAGE and stained with Coomassie Blue. (B) FACS analysis of intracellularly stained tumor cell lines with K94 antibody. (C) Western blot analysis of target antigen of K94 autoantibody. Total cell lysates (CL, 50 *μ*g) or concentrated cell culture media (CCM, 50 *μ*g) were resolved on 8–10% SDS-PAGE gels, followed by blotting and probing with K94 autoantibody. (D) Fractions enriched with K94 autoantigen (described in the Materials and methods, in detail) were pooled, concentrated and resolved on a 10% SDS-PAGE gel. Two protein bands corresponding to K94 autoantigen confirmed by immunostaining with K94 antibody were excised and analyzed by proteomic methods.

The isotype of K94 autoantibody was determined as IgM (data not shown) and K94 antibody was purified from K94 hybridoma supernatant or ascites fluid from mice inoculated with K94 hybridoma using protein L-agarose (Fig. 1A). The expression of K94 target antigen in various tumor cell lines was examined using purified K94 antibody. When examined by flow cytometric analysis of intracellularly stained cells (Fig. 1B), the reactivity of K94 autoantibody was significantly higher in several tumor cell lines (HepG2, SNU638, MCF-7 and A2780) than in non-tumor cell lines (Chang and HT22). This suggests that the expression of K94 target antigen might be related to tumorigenesis in various tissues such as liver. However, western blot analysis of K94 target antigen gave a different result (Fig. 1C). Although detectable in most tumor cell lines, K94 target antigen was detected most highly in HT-29 and MCF-7 cell lines rather than in hepatoma cell lines. Secretion of K94 antigen was also examined. In the process of immune response to self antigens, antigens must be exposed to immune cells in extracellular space. To confirm the secretion of K94 autoantigen from tumor cells, serum-free cell culture media from each cell line were collected, concentrated and analyzed by western blotting. K94 autoantigen was detected in serum-free media from MCF-7 breast cancer cells, but not from HT-29 or hepatoma cells (Fig. 1C).

### Identification of K94 target antigen

To identify the target antigen of K94 tumor-associated autoantibody, 500 ml of serum-free media from MCF-7 cells was collected, concentrated, and fractionated by HitrapQ ion exchange chromatography. The K94 antigen-enriched fractions were identified by western blot analysis, pooled and concentrated again (data not shown). Ninety percent of the concentrate was separated on a 10% SDS-PAGE gel, and stained with Coomassie Blue. The remaining concentrate was used for western blot analysis. Two protein bands from the Coomassie Blue stained gel, which were reactive to K94 antibody, were excised and treated with trypsin for in-gel digestion (Fig. 1D). The peptide extracts from in-gel digestion were then analyzed by Nano-LC-ESI-MS/MS, and two cytokeratins, CK8 and CK18, were identified from these extracts (Table II).

**Figure 2.**
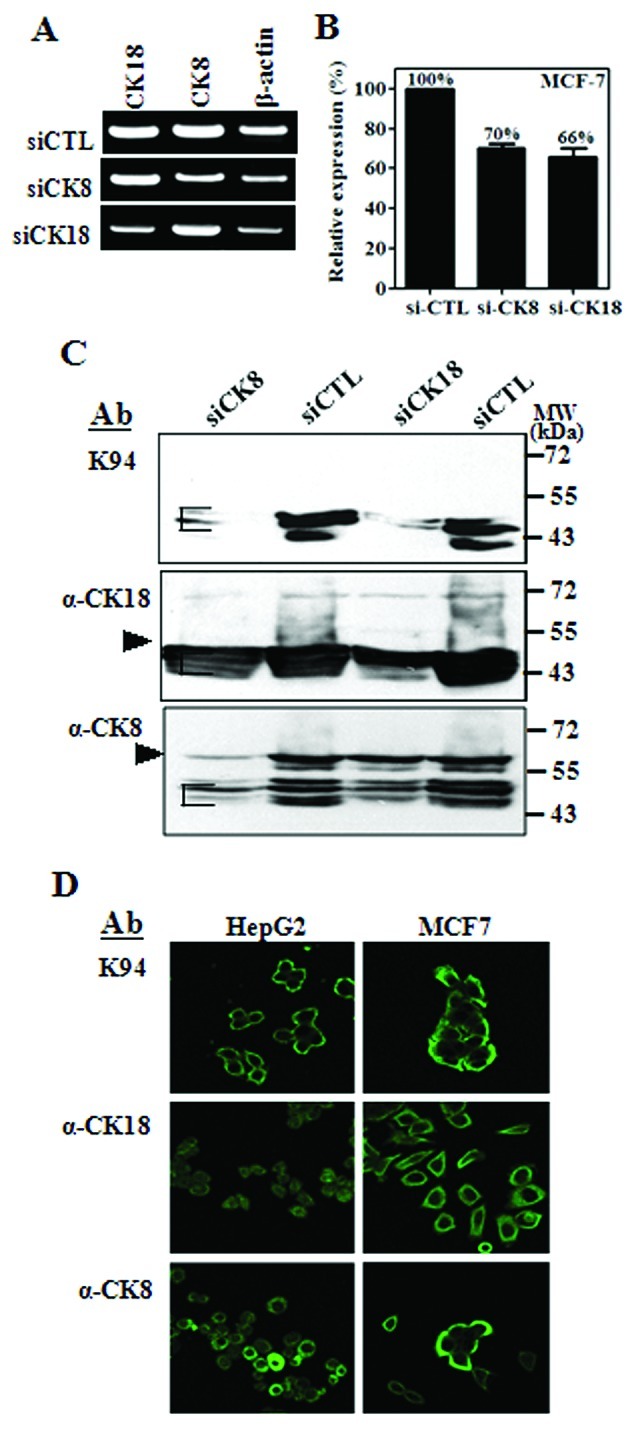
Validation of the CK8/CK18 complex as the K94 antigen using siRNA to CK8 or CK18. (A) RT-PCR analysis of CK8 or CK18 expression in MCF-7 cells treated with siRNA against CK8 or CK18. (B) FACS and (C) western blot analysis of MCF-7 cells treated with siRNA targeting CK8 or CK18 and stained with K94 antibody. Western blots were also probed with anti-CK8 or CK18 antibody. (D) Immunofluorescence staining of HepG2 and MCF-7 cells with K94 antibody.

CK8 is a 53-kDa intermediate filament protein composed of 483 amino acids, and is co-expressed with complementary cytokeratin protein, CK18, a 48-kDa protein composed of 430 amino acids (14). Protein bands probed with K94 antibody had a molecular weight of about 43 kDa, which are smaller than the full-length size of CK8 or CK18. To confirm the results of mass spectrometric analysis, MCF-7 cells were transfected with siRNA targeting CK8 or CK18 individually, and examined by flow cytometric analysis of intracellularly stained cells. RT-PCR results confirmed that the expressions of CK8 or CK18 were not influenced by suppression of complementary CK, although CK8 and CK18 form co-complexes in intermediate filaments (Fig. 2A). However, K94 antigens in intracellularly-stained cells were diminished in both cases of siRNA targeting (Fig. 2B). This suggests that the epitope specific to K94 antibody is influenced by the expression of both CKs. Western blot analysis demonstrated this phenomenon more precisely. As shown in Fig. 2C, the protein bands stained with K94 antibody were decreased in cases of both CK8 suppression and CK18 suppression. However, the protein bands stained with anti-CK8 or anti-CK18 antibody were decreased only when siRNA corresponding to each CK was treated. In addition, the protein bands reactive to anti-CK8 or anti-CK18 antibody were detected at molecular masses corresponding to their full size (CK8 at 55 kDa and CK18 at 43 kDa) and smaller sizes, which might be truncated forms of CK8 or CK18. However, the protein bands corresponding to K94 antigens had molecular weights of 43–40 kDa range, in which truncated CK18 and CK8 are overlapped and can form partial complex of CK8 and CK18. These results suggest that K94 antibody recognizes an epitope presented by complexing of CK8 and CK18.

### Intracellular localization of K94 autoantigen in tumor cell lines

To evaluate the cellular distribution of K94 autoantigen, which may be a CK8/18 complex, compared with CK8 or CK18 alone, tumor cell lines (HepG2 and MCF-7) were stained with K94, anti-CK8 and anti-CK18 antibodies and then examined by using confocal microscopy (Fig. 2D). In these cells, K94 antibody staining was most prominent near the plasma membrane, whereas individual staining of CK8 or CK18 was throughout the cytoplasm or nucleus. These patterns of K94 antibody staining were also observed in Hep3B, A549 and HT-29 cells (data not shown). These results are also consistent with the previous reports on CK8/18 which is distributed in cytoplasmic filament networks and as bands associated with the plasma membrane (15).

### Validation of CK8/18 complexes as the K94 autoantigen using recombinant CKs

**Figure 3.**
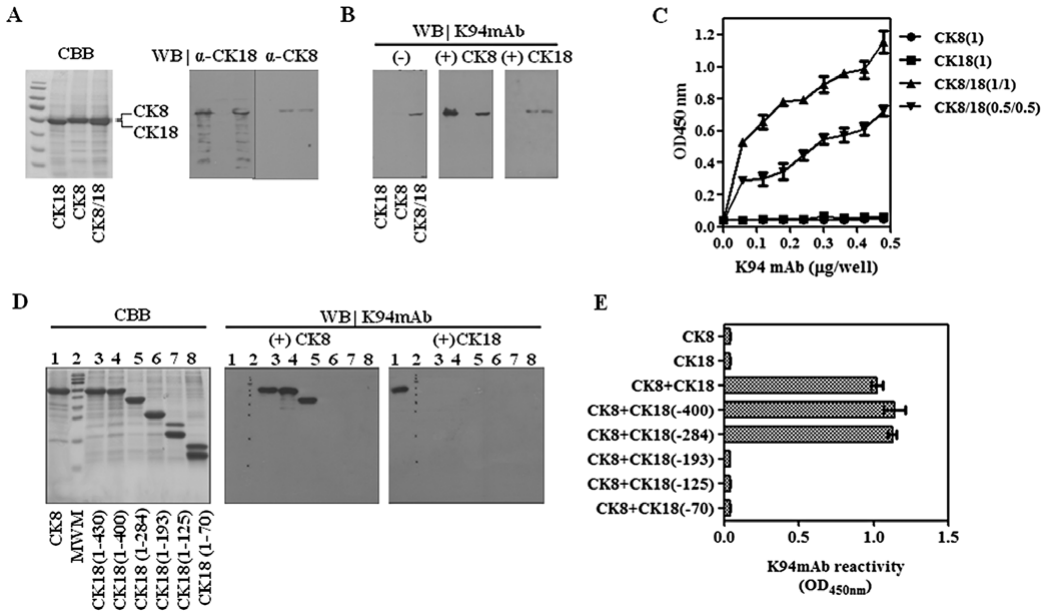
Validation of CK8/18 complexes as the K94 antigen using recombinant CKs. (A) Recombinant CK8 containing His- and S-tag (54.8 kDa), CK18 containing His-tag (53 kDa) and their mixtures were resolved on 10% SDS-PAGE and probed with specific antibodies (anti-CK8 or CK18 antibody). (B) CK8/CK18 heterotypic complexes were formed on PVDF membranes as described in Materials and methods and probed with K94 antibody. (C) CK8/CK18 heterotypic complexes formed by mixing CK8 (0.5 or 1 *μ*g/ml) and CK18 (0.5 or 1 *μ*g/ml) solutions were coated onto ELISA plates and probed with K94 antibody (described in Materials and methods, in detail). The epitope for the K94 antibody on CK8/CK18 heterotropic complexes was analyzed in detail using a series of truncated CK18 and full-length CK8 proteins on (D) PVDF membranes and (E) using ELISA.

Results of siRNA targeting demonstrate that K94 autoantibody recognizes an epitope presented by complexing of CK8 and CK18. To define the epitope recognized by K94 antibody, recombinant CK8, CK18 and their complexes were prepared. Recombinant CK8 and CK18 proteins were expressed using pET29a vector conferring the S-tag and His-tag, which increased their molecular weights to 54 and 53 kDa, respectively. Each protein or their mixtures were separated on 10% SDS-PAGE gels (Fig. 3A) and analyzed by western blots using K94 antibody (Fig. 3B). As expected, K94 had no reactivity to CK8 or CK18 protein, separately. However, the mixture of CK8 and CK18 showed reactivity to K94. Although CK8 and CK18 proteins were resolved on 10% SDS-PAGE gels, their co-localization on the blot seems to promote the partial formation of CK8/18 complexes. A sure method which allows formation of the CK8/CK18 complex was performed using immunoblots of CKs (13). After transferring CKs onto PVDF membranes and blocking with skim milk solution, the blots were incubated with complementary CKs to form CK8/CK18 heterotypic complexes, and then were probed with K94 autoantibody. As expected, K94 was only reactive to CK8/18 heterotypic complex (Fig. 3B). These results were also confirmed by ELISA. Recombinant CK8 was mixed with recombinant CK18 in a molar ratio of 1:1 in urea. The samples were then diluted with PBS to allow the formation of the heterotypic complex and coated on ELISA plates. In accordance with the results from western blot analysis, K94 antibody bound to CK8/18 complex, but not to CK8 or CK18 only (Fig. 3C). For a more detailed epitope mapping of K94 antibody, five truncated CK18s were prepared and CK8/CK18 complexes were formed on western blots of CK8 as described above, followed by probing with K94. Among the truncated CK18 proteins, CK18 (1–400) and CK18 (1–284) formed CK8/CK18 complexes displaying the epitope of K94 autoantibody (Fig. 3D). These results suggest that a conformational structure formed by CK8 and CK18 (194–284) is the specific antigenic determinant of K94 autoantibody. These results were also confirmed by ELISA (Fig. 3E).

### Mimotope screening

Autoantibody signatures captured by using auto-antigenic mimotope-containing phages, have been suggested as a potential diagnostic method for cancer screening. Wang *et al* screened a phage display cDNA library derived from prostate cancer tissue with patients’ sera, and successfully used their selected mimotope phages to measure tumor-associated autoantibodies for tumor diagnosis (16). Their results indicate that the antigenicity of the autoantigen is restricted to one or two epitopes of a target protein, which makes it possible to detect specific autoantibodies with only these antigenic structures without using whole antigen proteins. The restriction of antigenicity of a certain auto-antigen to only one epitope was also shown in the case of GRP78 (17). In our previous study, an autoantibody related to hepatocellular carcinoma was identified, of which specific target was fatty acid synthase (FASN) with a molecular weight of ∼200 kDa. The large size of FASN made it difficult to prepare recombinant FASN protein, which was necessary to formulate a detection method of anti-FASN autoantibody. To overcome these limitations, antibody-specific mimotopes were screened using a cyclic peptide display phage library and used successfully for the diagnosis of hepatocellular carcinoma (10).

**Figure 4.**
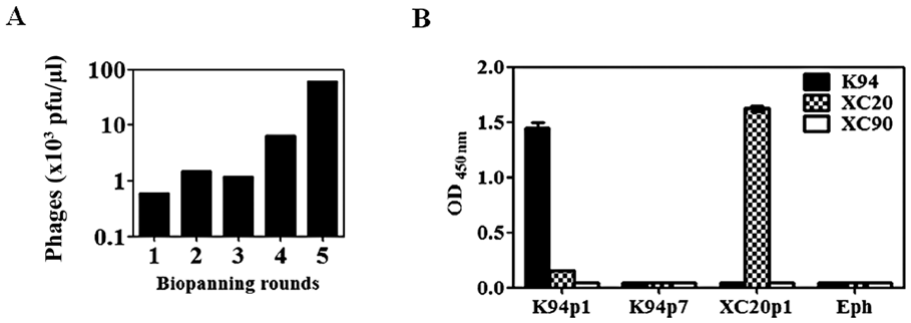
Selection of mimotope peptide specific for K94 antibody. (A) Panning of mimotope phage against K94 autoantibody. Five rounds of biopanning were performed against a random cyclic heptapeptide display phage library. (B) The reactivity of selected phages against K94 antibody was examined by phage ELISA. Empty phage (Eph) without an insert peptide sequence and XC20p1 phage were used as control antigens and other HCC-derived auto antibodies (XC20 and XC90) were used as control antibodies.

In this study, the specific binding site of K94 autoantibody was determined as a conformational structure formed by the heterotypic association of CK8 and CK18. To develop a detection method for CK8/CK18 complex recognition by auto-antibodies similar to K94 antibody, preparation of CK8/CK18 complexes would be a critical problem to solve, but not easy. Therefore, we determined the antigenic structure recognized by the K94 autoantibody using the peptide display phage library, which would be convenient bait for the detection of antibody. After five rounds of biopanning of the phage library (Fig. 4A), two phages were obtained having different inserted peptide sequences, K94p1 (CISPDAHSC) and K94p7 (CTLSHTRTC). The inserted peptide sequences from 9 out of 10 phages were identical to that of K94p1, and only one phage displayed the peptide sequence of K94p7. Their reactivities against K94 antibody were analyzed by ELISA. As shown in Fig. 4B, K94p1 mimotope phage showed high reactivity to K94. Other phages expressing peptide sequences of CTLSHTRTC (K94p7), CLSIGMPGC (XC20p1), or phage without the insert peptide sequence (Eph) showed no binding to K94.

### Human serum ELISA for the detection of autoantibody against CK8/18 complex

**Figure 5.**
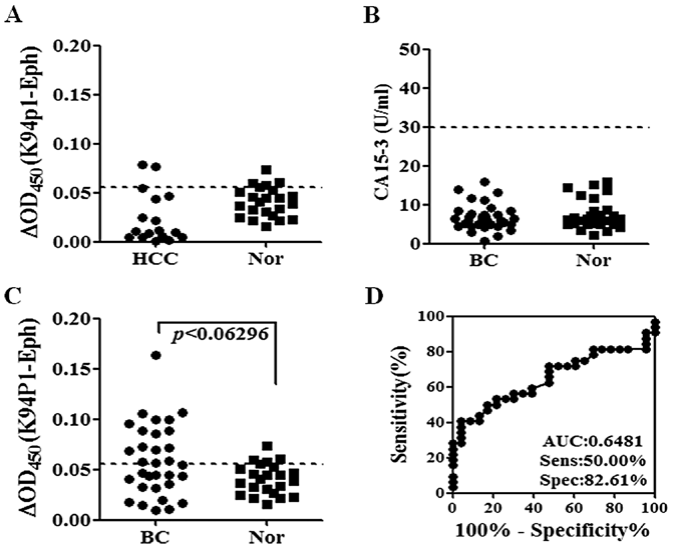
Human serum ELISA for the detection of autoantibody against CK8/CK18 complexes. (A) Detection of autoantibody against CK8/CK18 complexes in HCC patients’ sera by K94p1 phage ELISA. (B) Detection of CA15.3 in breast cancer patients’ sera using commercialized kits (eBioscience, San Diego, CA). (C) Detection of autoantibody against CK8/CK18 complexes in breast cancer patients’ sera by K94p1 phage ELISA. The specific binding of autoantibody against K94p1 phage was expressed as the difference between the OD value of K94p1 ELISA and that of empty phage (Eph) ELISA. These experiments were repeated three times and a representative result is shown. (D) ROC curve of K94p1 phage ELISA in Fig. 5C.

The K94p1 cyclic peptide mimotope expressed on M13 phage showed high specificity to K94 antibody, which can be used as bait for the detection of anti-CK8/18 antibody instead of recombinant CK complexes. K94 antibody is a tumor-associated autoantibody derived from mouse model of HCC. However, its reactivity to human tumor cell lines suggests the possibility of occurrence of autoantibodies with similar reactivity to K94 antibody in tumor patients’ sera, as in the case of anti-FASN autoantibody (10). Phage ELISA using the K94p1 phage as coating antigen was performed for the detection of autoantibody against CK8/18 complexes in human patients’ sera. First, the reactivity of hepatoma patients’ sera was examined. K94p1 phage was coated onto 96-well Maxisorp plates and after blocking, human sera pre-adsorbed with cell extracts from the phage host were treated as primary antibody, as described previously (18). The reactivity to hepatoma sera was not different from that of normal sera (Fig. 5A). The existence of autoantibodies in breast cancer patients’ sera was also examined because K94 antigen was overexpressed and secreted from MCF-7 breast cancer cells, as shown in Fig. 1C. Sera were obtained from breast cancer patients in stages 0 to 3. Their CA15.3 levels were far below 30 U/ml, which is a cutoff value distinguishing breast cancer patients from normal subjects (19), and differences between breast cancer and normal subjects were not found (Fig. 5B). However, K94p1 phage ELISA discriminated breast cancer patients from normal subjects, as shown in Fig. 5C. The sensitivity of this assay was 50% and specificity was 82.61% (Fig. 5D). Clinical information of breast cancer patients (n=30) were also analyzed for the levels of anti-CK8/18 autoantibody measured by K94p1 phage ELISA (Table III). However, there were no significant differences between anti-CK8/CK18 positive and anti-CK8/CK18 negative groups with respect to factors analyzed in this study.

At present, mimotope ELISA composed of K94p1 peptide is not so sensitive for the diagnosis of breast cancer, although more useful than CA15.3 assay. However, these results show a possibility of its usage as a simple diagnostic method of breast cancer only using patient sera instead of imaging techniques.

## Discussion

Breast cancer is the most common invasive cancer in woman. Mammography is a widely used primary screening method for breast cancer, however, it has only moderate sensitivity (20,21) and specificity (22). Several serum protein markers for breast cancer have been suggested, such as CA15.3 and Her-2. However, these markers are also not sensitive enough for screening and early diagnosis. To overcome these limitations recent studies on breast cancer biomarkers have suggested the use of autoantibody biomarkers, which are detectable in patients’ sera even when the tumor is in an early stage (23–26). Autoantibody to aberrantly glycosylated MUC1, in early-stage breast cancer, is a typical example of such cases. Assays to detect the cancer-associated antigen CA15.3 (also known as MUC1) in serum are widely used for monitoring disease progression and response to therapy in some late-stage breast cancer patients. But this assay does not detect elevated levels of MUC1 in serum from patients with early-stage diseases. In contrast to MUC1 antigen, autoantibodies to aberrantly glycosylated MUC1 are found more frequently and at higher levels in early-stage breast cancer patients (23). It may be useful as a possible early diagnostic and prognostic marker of breast cancer. Detection of autoantibody to MUC1 was, however, not successful when unglycosylated MUC1 or undefined glycoforms of MUC1 were used as autoantibody antigens (27). These results suggest again that a unique antigenic determinant, not a whole antigen, is important for the induction of autoantibody and for determination of an appropriate mimotope structure.

In this study, a tumor-associated autoantibody against a CK8/CK18 complex was identified as a breast cancer biomarker. The autoantibody against the CK8/CK18 complex, K94, derived from an HCC mouse model, was purified and its antigenic determinant was identified as a conformational epitope formed by complexing of CK8 and CK18. Autoantibody detection using recombinant CK8/CK18 protein was not effective (data not shown), which might be caused by inappropriate presentation of the conformational antigenic structure by a recombinant protein. To overcome these limitations, a cyclic peptide display phage library was screened to identify a mimotope structure, which was effective for the detection of anti-CK8/CK18 autoantibody in breast cancer sera.

CK8 and CK18 are the major components of intermediate filaments of simple or single layer epithelia. These filaments are found in the intestine, liver and breast duct (14), and coincides with the expression of K94 antigen in liver, colon and breast cancer cell lines as determined by western blot and flow cytometric analysis. CK18 gene expression is known to be stimulated by Ras signal transduction pathway proteins (28), and increased expression of CK8 and CK18 has been found at the invasive front of some tumors. These findings implicate CK8 and CK18 are related in the tumorigenic phenotype (29). In addition, an association between CK8 and CK18 expression, and increased invasiveness and metastatic properties through specific interactions with the extracellular environment, has been observed (30,31). The occurrence of anti-CK8/CK18 autoantibody K94 in the H-ras12V transgenic mouse suggests that CK8/CK18 over-expression in the mouse model might be induced by activation of Ras signaling and the release of CK8/CK18 complexes into the extracellular region be the cause of autoantibody response, although they were not analyzed in this study. These phenomena also may be relevant to breast cancer, where autoantibody against CK8/CK18 was detected.

The secretion mechanism of CK8/CK18 complexes from tumor cells is unclear. CK8 or CK18 have no classical secretory signal sequences and are not membrane-associated proteins. Early studies on CK8 or CK18 in sera suggested that apoptosis or necrosis of tumor cells or inflammatory cells are the main reasons for their presence in extracellular regions (32). However, recent studies on autoantigens suggest a non-classical secretory mechanism via microvesicles or exosomes (33–35). Cancer-associated cleavage of CK8/CK18 (13) and a non-classical secretory mechanism might be additional causes for extracellular CK8/CK18, which might be studied further.

Detection of CK8/CK18 complexes in patients’ sera was also assessed by western blot or dot blot analysis using K94. However, target antigen was not detectable in any sera (data not shown), which may be due to small amounts or instability of target antigen in the sera. These results again suggest that the immune system serves to amplify the detection of the antigen (36).

The potential use of autoantibody to the CK8/CK18 complex as a cancer biomarker was the purpose of our study. There is a report on a human anti-CK8/CK18 IgM designated as COU-1, which reacts with another conformational epitope on CK8/18 complex (37). COU-1 was purified from a human B-cell hybridoma derived from a colon cancer patient and recognized a unique conformational epitope presented only by a complex between CK8 and CK18. Its epitope was revealed after proteolytic removal of the head domain of either CK8 or CK18 and its specific antigen was highly expressed in colon, breast and ovarian cancer, which made it useful for immunostaining or therapy of cancer. In our view, COU-1 is an autoantibody induced in colon cancer patients that can be used as a tumor marker; however, it had not been considered as a potential cancer biomarker.

Our previous study on anti-FASN autoantibody derived from an HCC mouse model had suggested a novel diagnostic method for human HCC (10). By studying K94 autoantibody, we provide further evidence that studies on autoantibodies derived from tumor mouse models are useful for the studies of human tumor-associated autoantibodies. Further studies on autoantibodies derived from tumor mouse models might identify novel candidate autoantibodies recognizing unique epitopes and these specific epitopes might be useful for tumor-associated autoantibody diagnostic assays.

## Figures and Tables

**Table I.** Sequence of PCR primers used for cloning of CK8, CK18 and truncated CK18 proteins.

Target/primer orientation	Primer sequence
CK8	
Forward	ccg catatg ATG TCC ATC AGG GTG ACC
Reverse	ata gtcgac CTT GGG CAG GAC GTC AGA
CK18	
Forward	ccg gaattc ATG AGC TTC ACC ACT CGC
Reverse	ata ctcgag ATG CCT CAG AAC TTT GGT
CK18 (1–70)	
Forward	ccg gaattc ATG AGC TTC ACC ACT CGC
Reverse	ata ctcgag ACC CCC GGC TAT CCC GGT
CK18 (1–125)	
Forward	ccg gaattc ATG AGC TTC ACC ACT CGC
Reverse	ata ctcgag GTC TCT GAC CTG GGG TCC
CK18 (1–193)	
Forward	ccg gaattc ATG AGC TTC ACC ACT CGC
Reverse	ata ctcgag ATT GGT GTC ATC AAT GAC
CK18 (1–284)	
Forward	ccg gaattc ATG AGC TTC ACC ACT CGC
Reverse	ata ctcgag TGT GGT GAC CAC TGT GGT
CK18 (1–400)	
Forward	ccg gaattc ATG AGC TTC ACC ACT CGC
Reverse	ata ctcgag GTT GCT GCT GTC CAA GGC

**Table II.** Identification of K94 autoantigen by mass spectrometric analysis.

Band position	Identified proteins	Accession number	Molecular mass	Queries matched	Mascot score
1	KRT18 keratin, type I cytoskeletal 18	IP00554788	48029	69	1298
	KRT8 keratin, type II cytoskeletal 8	IP00554648	53671	56	931
2	KRT18 keratin, type I cytoskeletal 18	IP00554788	48029	100	2310
	KRT8 keratin, type II cytoskeletal 8	IP00554648	53671	67	1120

**Table III.** Clinical information and anti-CK8/18 reactivity in breast cancer patients.

Category	No. of patients	anti-CK8/18 n (%)		CA15-3 n (%)	
		<	> cutoff	<	>10
Age (years)					
≤50	13	8 (62)	5 (38)	10 (77)	3 (23)
>50	17	8 (47)	9 (53)	11 (65)	6 (35)
Stage					
0	2	1 (50)	1 (50)	1 (50)	1 (50)
I	10	5 (50)	5 (50)	8 (80)	2 (20)
IIA	7	4 (57)	3 (43)	5 (71)	2 (29)
IIB	8	4 (50)	4 (50)	6 (75)	2 (25)
IIIA	3	2 (67)	1 (33)	1 (33)	2 (67)
Alkaline phosphatase (IU/l)					
≤140	7	4 (57)	1 (14)	4 (57)	1 (14)
>140	23	12 (52)	11 (48)	15 (65)	8 (35)
CA15-3 (IU/l)					
≤10	21	12 (57)	9 (43)	-	-
>10	9	4 (44)	5 (56)	-	-
Tumor size (cm)					
≤2	9	4 (44)	5 (56)	7 (78)	2 (22)
>2	21	12 (57)	9 (43)	15 (71)	6 (29)
Pathology					
Invasive duct carcinoma	22	13 (59)	9 (41)	15 (68)	7 (32)
Others^a^	8	3 (38)	5 (63)	6 (75)	2 (25)
Estrogen receptor status					
−	6	4 (67)	2 (33)	5 (83)	1 (17)
+/−	3	0 (0)	3 (100)	2 (67)	1 (33)
++	6	4 (67)	2 (33)	6 (100)	0 (0)
+++	14	8 (57)	6 (43)	7 (50)	7 (50)
Progesterone receptor status					
−	9	5 (56)	4 (44)	7 (78)	2 (22)
+	5	2 (40)	3 (60)	3 (60)	2 (40)
++	2	2 (100)	0 (0)	1 (50)	1 (50)
+++	12	8 (67)	4 (33)	8 (67)	4 (33)
p53					
−	5	1 (20)	4 (80)	3 (60)	2 (40)
+	15	11 (73)	4 (27)	13 (87)	2 (13)
++	2	2 (100)	0 (0)	1 (50)	1 (50)
+++	5	2 (40)	3 (60)	3 (60)	2 (40)
erbB-2					
−	20	11 (55)	9 (45)	14 (70)	6 (30)
+++	8	5 (63)	3 (38)	6 (75)	2 (25)

a Others, invasive lobular carcinoma, invasive mucinous carcinoma, medullary carcinoma.
